# Numerical Evaluation of Combustion Regimes in a GDI Engine

**DOI:** 10.1007/s10494-018-9949-8

**Published:** 2018-06-19

**Authors:** N. J. Beavis, S. S. Ibrahim, W. Malalasekera

**Affiliations:** 1 Department of Aeronautical and Automotive Engineering, Loughborough University, Loughborough, Leicestershire LE11 3TU UK; 2 School of Mechanical and Manufacturing Engineering, Loughborough University, Loughborough, Leicestershire LE11 3TU UK

**Keywords:** Numerical, CFD, Combustion, Turbulence, GDI, Engine

## Abstract

There is significant interest in the gasoline direct-injection engine due to its potential for improvements in fuel consumption but it still remains an area of active research due to a number of challenges including the effect of cycle-by-cycle variations. The current paper presents the use of a 3D-CFD model using both the RANS and LES turbulence modelling approaches, and a Lagrangian DDM to model an early fuel injection event, to evaluate the regimes of combustion in a gasoline direct-injection engine. The velocity fluctuations were investigated as an average value across the cylinder and in the region between the spark plug electrodes. The velocity fluctuations near the spark plug electrodes were seen to be of lower magnitude than the globally averaged fluctuations but exhibited higher levels of cyclic variation due to the influence of the spark plug electrode and the pent-roof geometry on the in-cylinder flow field. Differences in the predicted flame structure due to differences in the predicted velocity fluctuations between RANS and LES modelling approaches were seen as a consequence of the inherently higher dissipation levels present in the RANS methodology. The increased cyclic variation in velocity fluctuations near the spark plug electrodes in the LES predictions suggested significant variation in the relative strength of the in-cylinder turbulence and that may subsequently result in a thickening of the propagating flame front from cycle-to-cycle in this region. Throughout this paper, the numerical results were validated against published experimental data of the same engine geometry under investigation.

## Introduction

### Background

Ever increasingly stringent emissions legislation for improved air quality and a need to reduce CO_2_ emissions and energy requirements to address the growing concern over our impact on climate change, provide motivation for the continued pursuit of increased understanding and optimisation of the internal combustion engine (ICE).

Investigations into the physical processes occurring within the ICE have been of research interest for a number of decades, but in spite of this continued effort, the complexity of the physical processes involved, the difficulty of non-intrusive access with experimental investigation, and the limitations in computing resource for detailed numerical investigation, mean our understanding of the physical processes within ICE’s still continues to develop.

Experimental techniques, whilst are common place, have their limitations, particularly with respect to their ease of measuring turbulence characteristics across all three spatial planes. The improvements in computational resource over the last decade have allowed increasingly complex numerical techniques to be pursued within ICE research, including the use of Large Eddy Simulation (LES) for modelling turbulence effects to allow both the anisotropic characteristics and cycle-by-cycle variations (CCV) in the flow field to be predicted. The former being particularly challenging to measure experimentally and the latter being an area of high research effort due to its effect on fuel consumption, emissions and driveability [1].

The use of diagrams to depict the regimes of turbulent combustion by the use of non-dimensional characteristic numbers have been proposed by a number of authors including Abraham, Williams and Bracco [2], Borghi [3] and Peters [4]. Whilst also aiding our understanding of the regimes of turbulent premixed combustion, these diagrams are also essential for assisting in the development of turbulent combustion models. Even during the earliest proposal of such diagrams, a region where turbulent premixed flames within ICE’s were expected to fall was identified but even to this day, uncertainty still exists in the range of expected operation within ICEs.

### Present contribution

The present study aimed at utilising a numerical approach for furthering our understanding of the turbulence characteristics and premixed combustion regime in a gasoline direct-injection (GDI) engine.

The main objectives were to utilise the LES approach to model the in-cylinder turbulence and a Lagrangian discrete droplet model (DDM) to model an early-injection direct injection event, to characterise the velocity fluctuations within the cylinder at the point of spark timing. Then, using published experimental results, combine the predicted velocity fluctuations with estimates of the integral length scale and laminar flame speed and thickness to predict the expected regime of combustion on the turbulent premixed combustion diagrams of [2] and [3]. Comparisons are made between the numerical predictions and published experimental data and on the predicted CCV of the combustion regime due to CCV in turbulence. Numerical predictions using a RANS k-*ε* turbulence model are also shown to indicate the impact on predictions when using a turbulence model with an isotropic turbulence assumption.

To the best of the authors’ knowledge, this is the first time that the LES turbulence modelling approach and a Lagrangian DDM have been applied to characterise velocity fluctuations at spark timing, and then combined with combustion regime diagrams to predict the resultant flame structure in an early-injection GDI engine.

## The Numerical Model

### The research engine

**Fig. 1:**
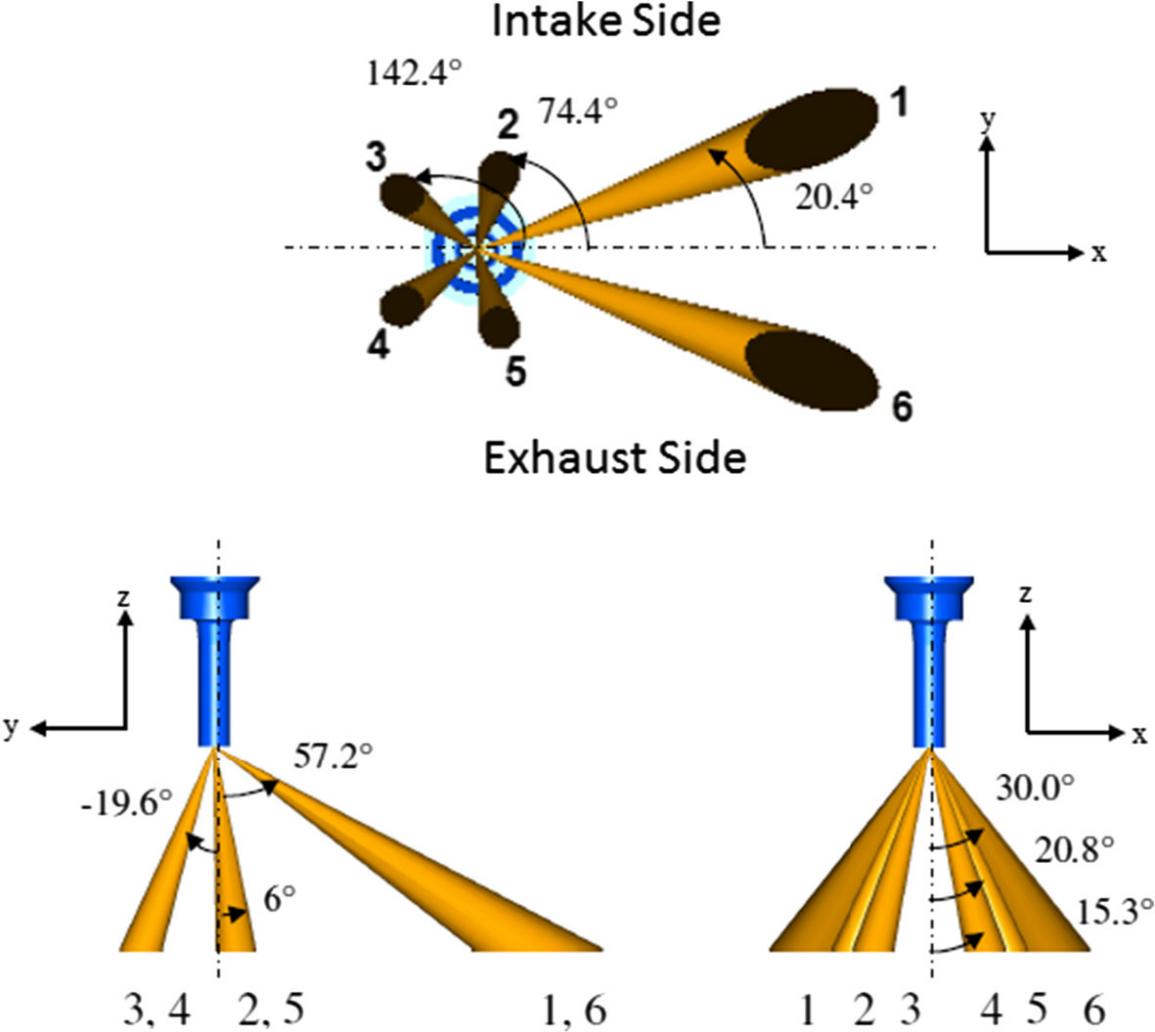
Spray plume orientation, reproduced from [6]

The engine that was the subject of this research is a single cylinder four stroke optical research engine based on the combustion chamber of a V8 engine with pent-roof cylinder head, flat piston crown and four valves per cylinder, representative of a typical commercial GDI engine design, as summarised in Table 1. The injector was a vertically and centrally mounted six-hole injector with y-plane plume symmetry. The spray plume orientation is shown in Fig. 1.

**Table 1:** Summary of optical research engine [5]

Bore	89 mm
Stroke	90.3 mm
Conrod length	148.97 mm
Compression ratio	10.5:1 nominal
Intake valve cam opening	24 ^∘^ ATDC
Intake valve cam closing	274 ^∘^ ATDC
Exhaust valve cam opening	224 ^∘^ ATDC
Exhaust valve cam closing	6 ^∘^ ATDC

### The computational domain

**Fig. 2:**
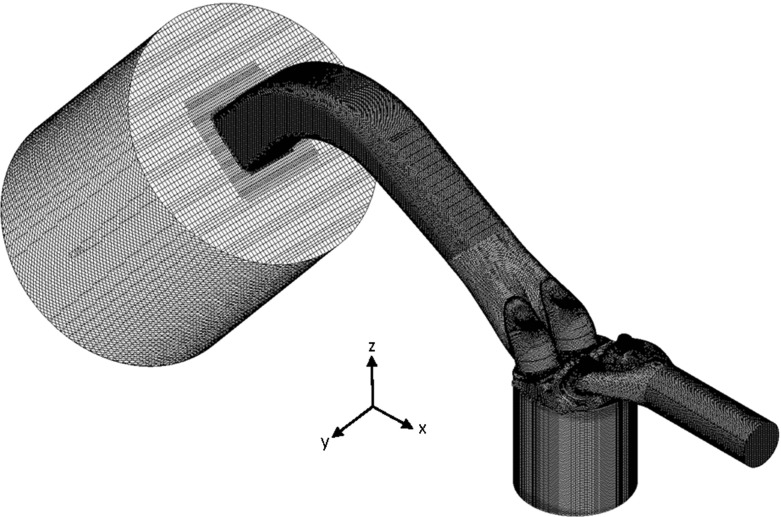
Computational mesh

The numerical model was developed using CFD code STAR-CD (v4.22) and was developed as a detailed representation of the experimental engine configuration. The computational domain is shown in Fig. 2. The domain was extended both upstream and downstream to allow sufficient time for turbulence to develop prior to the cylinder and to prevent recirculating flow around the flow outlet, respectively. The mesh contained approximately 2.2million cells at Bottom Dead Centre (BDC) and had a typical cell size across the cylinder of approximately Δ*x* = 0.8 mm in each spatial direction.

**Fig. 3:**
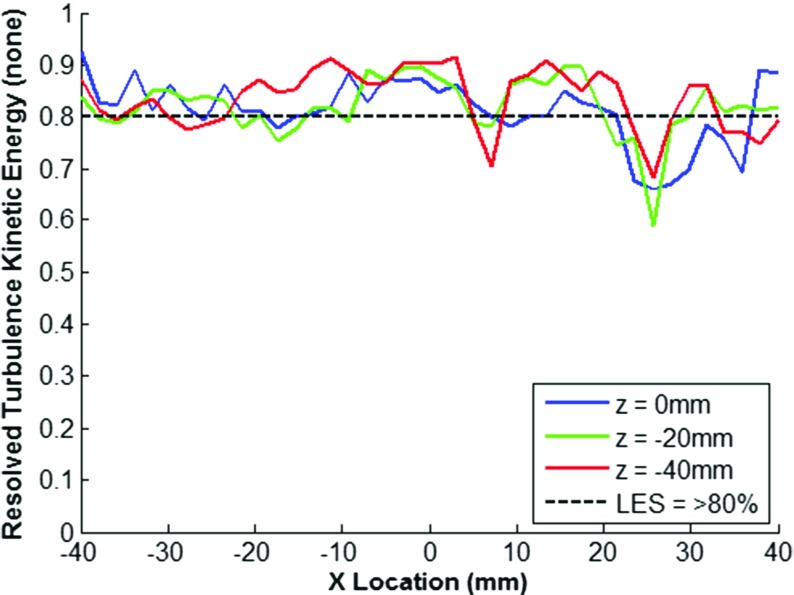
Resolution of Turbulence Kinetic Energy as a function of X-axis Position at 510^∘^ c.a

**Fig. 4:**
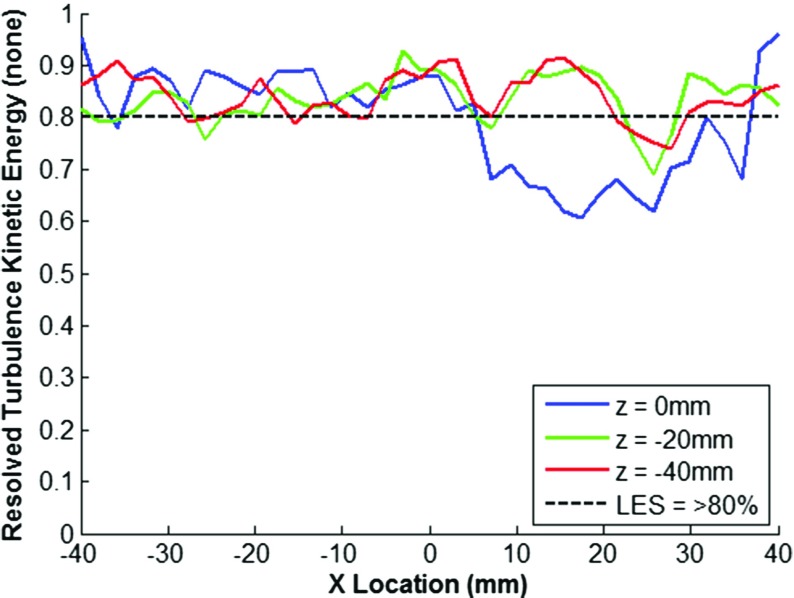
Resolution of Turbulence Kinetic Energy as a function of X-axis Position at 540^∘^ c.a

**Fig. 5:**
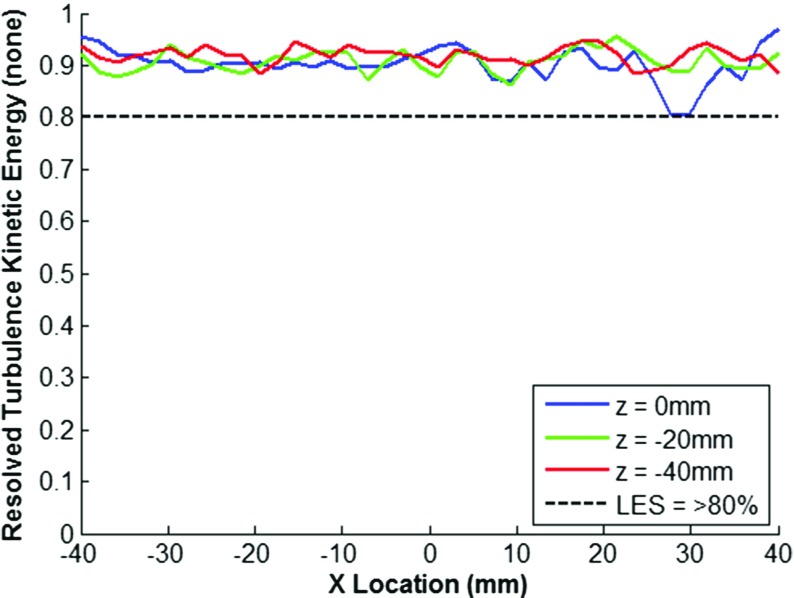
Resolution of Turbulence Kinetic Energy as a function of X-axis Position at 630^∘^ c.a

In LES simulations, the solution is dependent on the filter width, thus the mesh suitability to capture the length scales present within the flow field, and hence its ability to capture a sufficient quantity of the flow turbulence kinetic energy, is not known *a priori* with a non-solution adaptive gridding approach. In this study, the turbulence resolution parameter as defined by Eq. 1, originally proposed by Pope [7], has been used to assess the suitability of the mesh at three different crank angles through the intake and compression strokes, along three swirl cutting planes (z-axis), as shown in Figs. 3, 4 and 5.

1 $$ M_{res} (x,t)=\frac{k_{res} (x,t)}{k_{res} (x,t)+k_{sgs} (x,t)} $$

The results show that generally turbulence resolution (*M*(*x*,*t*)) is greater than 80% which is considered with a small degree of deviation down to approximately 60% resolution at earlier crank angles due to insufficient resolution of the high shear regions around the intake valve jet. Values of *M*_*res*_(*x*,*t*) > 80% are considered as a requirement to be deemed a ‘Large’ eddy simulation [8], whereas values between 60%-80% are considered a ‘Very Large’ eddy simulation. In the interests of maintaining a reasonable computational expense, the mesh was deemed acceptable for sufficient resolution of the in-cylinder flow field and no further mesh refinement was pursued.

### Boundary and initial conditions

The modelled operating condition was based on a standardised operating condition, typical of a low speed inner city driving condition, with the caveat of a modified liner coolant temperature that was used to drive increased liner wetting in the operating condition that the model was validated against (Table 2).

**Table 2:** Summary of the operating condition and numerical boundary conditions

Engine Speed	1500 rpm
Engine Load / BMEP	2.6 bar
Fuel type	Iso-octane
Fuel temperature	363 K
Injection timing	80^∘^ ATDC
Spark timing	35^∘^ BTDC
Injection pressure	150 bar
Pulse width	0.78 ms
Fuel-air equivalence ratio	1
iEGR (determined by valve timing strategy)	˜15%
Inflow gas pressure (abs)	0.453 bar
Inflow gas temperature	301 K
Inflow turbulence	Intensity: 0.1 Length scale: 4.8mm
Outflow gas pressure	1.023 bar
Outflow gas temperature	784 K
Outflow turbulence	Intensity: 0.1 Length scale: 1mm
Cylinder liner temperature	293 K
Cylinder head temperature	363 K
Piston crown temperature	301 K
Intake valve temperature	323 K
Exhaust valve temperature	363 K

Both RANS and LES turbulence model simulations were initialised by first running a complete RANS cycle, and the LES model was run for a further LES cycle, to adequately establish the correct prediction of intake system wave dynamics and minimise the influence of initial conditions on the in-cylinder numerical predictions.

### Computational setup

Spatial discretisation was achieved by a combination of second-order schemes, dependent on the discretised scalar. The second order accurate differencing scheme Monotone Advection and Reconstruction Scheme was used for turbulence kinetic energy, turbulence dissipation, momentum and energy equations. The Central Differencing scheme was used for density. Temporal discretisation was achieved by the Pressure Implicit with Splitting of Operator pressure-correction algorithm [9, 10] which results in temporal accuracy of approximately second-order, with a moderate amount of pressure under-relaxation to further improve solver stability.

The time-step was set at 5.6× 10^− 6^ s (equating to approximately 0.05ca/time-step) except around valve opening and closing periods where it was set to 1.1x10^− 6^s (approximately 0.01ca/time-step). This provided adequate solution stability, an average Courant–Friedrichs–Lewy (CFL) number of less than one and a solver time of approximately 3 days per complete engine cycle.

### Turbulence modelling

#### The LES SGS turbulence model

As part of the numerical predictions used in this study, the LES approach is applied where above a certain filter width the Navier-Stokes equations are solved directly for the large scales using space-filtered equations and below a certain filter width the small scales are modelled using a sub-grid scale (SGS) model. In this study the Smagorinsky [11] SGS model is used. This particular SGS model was used due to not using any additional space filtering or transport equations hence reducing the computational cost and increasing solution instability. The Smagorinsky constant (*C*_*s*_) was set to 0.02 [12] and the filter width was defined by the cube root of the cell volume.

#### The RANS turbulence model

For comparative purposes, the RANS turbulence modelling approach is also used within this study where the Navier-Stokes equations are time-filtered and the resultant equations closed by a turbulence viscosity approach. In this study the RNG k-*ε* [13, 14] due to it more effectively accounting for the effects of compression, expansion and rapid strain on the turbulent scales and a number of studies showing positive results within ICE’s [15, 16].

#### Turbulence modelling near the wall

In both turbulence sub-model cases, turbulence in the near-wall region is modelled using the Angelberger [17] sub-model with constants set as follows: y$^{+}_{\text {sw}}=$13.2, a_w_ = 2.075, b_w_ = 3.9

### Fuel injection modelling

#### Fuel injection model inputs

**Fig. 6:**
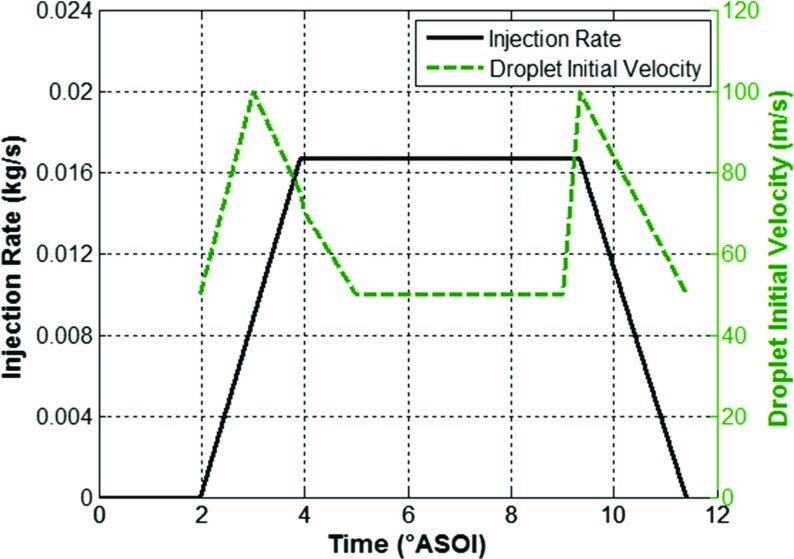
Computational injection rate profile and initial droplet velocity

The fuel injection event was modelled using a Lagrangian DDM with a summary of the inputs used shown in Table 3 and Fig. 6.

**Table 3:** Fuel injection inputs

Droplet Distribution	Rosin-Rammler:
	X = 14× 10^− 6^ m, q = 2.3
Number of Injected Parcels	50’000 parcels per jet
Droplet Initial Velocity	Shown in Fig. 6
Injection Rate	Shown in Fig. 6
Total Injected Mass	13.8 mg

A Rosin-Rammler distribution was used to provide the initial droplet size distribution, with the constant ‘q’ set to 2.3 based on the experimental works of [18] which used a similar injector configuration and experimental conditions and the constant ‘X’ set to 14× 10^− 6^ m which provided the best match against experimental Phase Doppler Anemometry (PDA) droplet size data.

A dependency study was completed to evaluate the influence of the number of injected parcels on the plume tip penetration and average droplet characteristics. The results showed that with the current mesh and sub-models, 50’000 parcels per jet provided a parcel number independent solution with acceptable computational expense.

The droplet velocity at the injector nozzle was imposed via a time-dependent profile as shown in Fig. 6, with an increase in initial droplet velocity used around the needle opening and closing to provide the best match against experimental plume tip velocity data – results shown below in the section ‘2.6 Model Validation’.

#### Fuel injection sub-models

The sub-models used within the fuel injection model that are common between both RANS and LES turbulence modelling approaches are outlined in Table 4.

**Table 4:** Summary of physical sub-models and constants used

Breakup Model	Pilch & Erdman [22] [B1 = 0.375, B2 = 0.2274]
Collision Model	O’Rourke [23], with additional sub-models for algorithm speed-up [24], automatic coalescence timestep adjustment [25] and additional geometric constraints [26] [K_rm_ = 1]
Droplet-Wall Interaction Model	Senda et al. [27–31], Bai & Gosman [32], Rosa et al. [33] [c_f_ = 0.7]
Leidenfrost temperature determination	Habchi [34] & Spiegler [35]
Liquid Film Model	Bai & Gosman [36] [*γ*_c_ = 0.8]
Liquid Film Model – Boiling Model	White [37] [C_sf_ = 0.06, *n* = 3, C_S_ = 1.2, c$_{\max }=$0.15, c$_{\min }=$0.09]
Liquid Film Model – Film Stripping due to Flow Over Edge	Friedrich [38] [$\theta _{\min }=$45^∘^, FR_c_ = 1, c1 = 3.78, q = 1.5]
Liquid Film Model – Film Stripping due to Wave & Body-Force Induced Instability	Fourcart [39]
Liquid Film Model – Effect of Contact Angle	Fourcart [39] [*𝜃*_c_ = 35^∘^, c = 1]

The droplet turbulence dispersion sub-model differs between the turbulence modelling approach used, where the difference is summarised in Table 5 and discussed in more detail here.

**Table 5:** Droplet Turbulence Dispersion Differences between LES and RANS Approaches

RANS	Gaussian pdf [19] where mean velocity is taken from the time-averaged local flow velocity and *k* is taken from the *k*-equation
LES	Gaussian pdf [19] where mean velocity is the sum of the local filtered-velocity from the momentum equation and SGS velocity from SGS model, and *k* is calculated from the SGS velocity

The droplet turbulence dispersion is typically modelled via a stochastic approach [19], whereby the droplet instantaneous velocity is equal to the sum of the mean velocity and the fluctuating velocity where it is assumed that the fluctuating velocity within an eddy is isotropic and follows a Gaussian probability density function (pdf) and that the interaction time is assumed sufficiently short that the fluid velocity in an eddy is effectively constant. The Gaussian pdf has a mean of zero and a standard deviation as defined by Eq. 2 where *k* is the turbulence kinetic energy.

2 $$ \sigma =\sqrt {\frac{2}{3}k} $$

When using the RANS k-*ε* turbulence modelling approach, the mean velocity is taken from the time-averaged local flow velocity from the turbulence model and the turbulence kinetic energy is the modelled turbulence kinetic energy taken from the k-equation.

When using the LES turbulence modelling approach, the turbulent dispersion is used to represent turbulence effects at the SGS on the droplet position and velocity. Hence, in this context, the resultant droplet velocity uses the filter-velocity for the mean velocity component, and the standard deviation of the Gaussian pdf uses the SGS kinetic energy, either from a SGS kinetic energy equation (if it is present in the LES SGS model) or calculated from the SGS velocity. Thus the droplet relative velocity is now a function of the filtered-velocity and the turbulent fluctuations, imposed on the droplet from the continuous-phase as a function of the SGS velocity, including any anisotropic characteristics. Hence all droplet conservation equations (mass, momentum and energy) and subsequent sub-models (including break-up, droplet collision, impingement and liquid film) are also a function of both the filtered-velocity and the SGS velocity.

The above description considers the energy exchange from the continuous-phase to the dispersed phase but does not consider the converse where energy from the dispersed-phase is imparted on the continuous-phase. The droplets in this research have a diameter of the order of micrometres, whereas the smallest resolved scales of turbulence when using the LES approach are defined by the filter width, which is dependent on the cell size, hence are of the order of millimetres. Thus as the droplets lose momentum, much of their energy is transferred to the continuous-phase at the SGS. A methodology for the exchange of energy from the discrete-phase to the continuous-phase at the SGS when using a LES SGS model that does not include a turbulence kinetic energy transport equation, such as the Smagorinsky SGS model as used in this study, is not yet available and will see a subsequent over-prediction of droplet kinetic energy [20, 21]. Nevertheless, in spite of this inherent limitation in this approach, the predictions show good agreement with experimental results (as shown below in section ‘2.6 Model Validation’) thus the authors still see a benefit in pursing this approach in lieu of using a more complex LES SGS model.

### Model validation

**Fig. 7:**
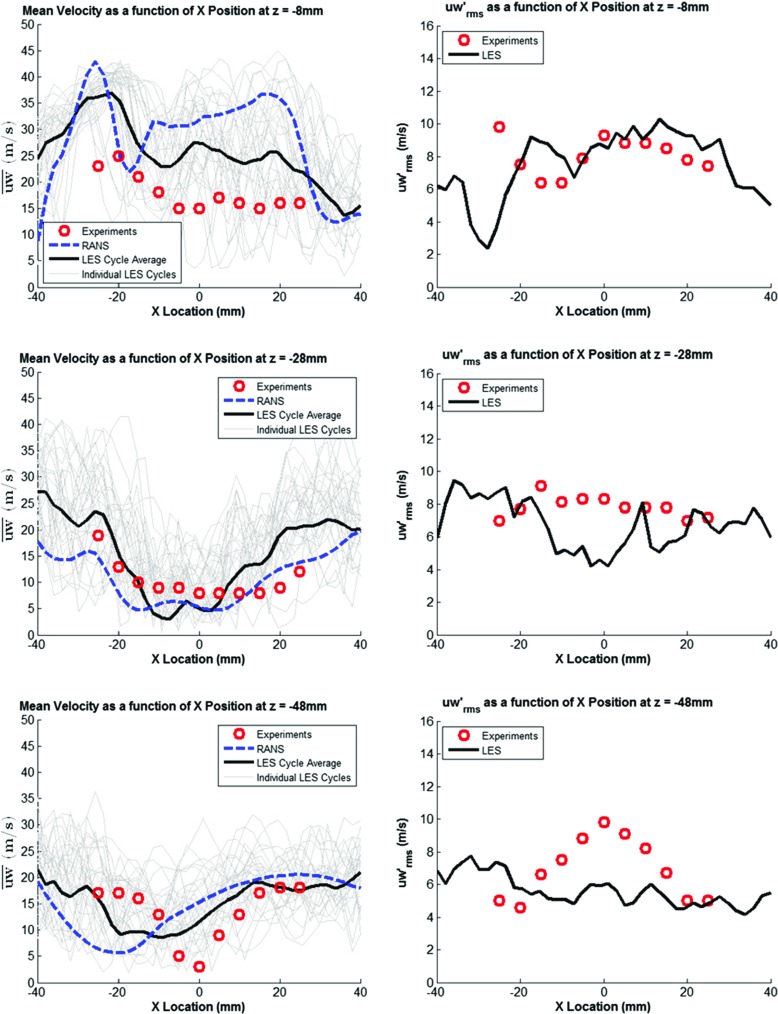
Mean and RMS Fluctuating Velocity at 100^∘^ ATDC

**Fig. 8:**
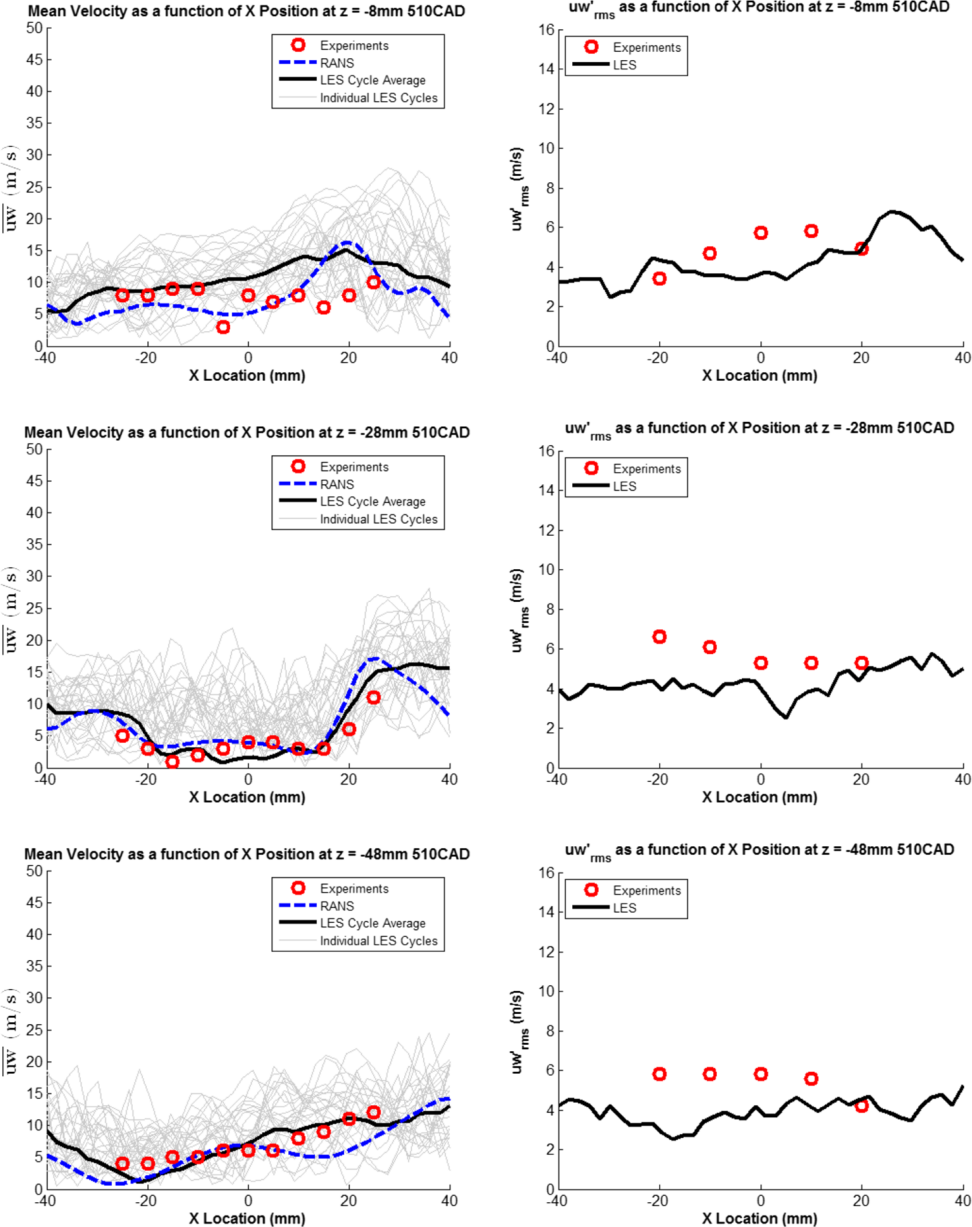
Mean and RMS Fluctuating Velocity at 150^∘^ ATDC

Before simulating the fuel injection event, the CFD models ability to correctly predict the in-cylinder flow field was evaluated as a single-phase simulation (i.e. cold flow). Results from the ensemble-average of 30 complete LES engine cycles with the Smagorinsky SGS turbulence model and a RANS cycle with the RNG k-*ε* turbulence model were compared against experimental results for mean velocity (*ū*) and root mean square of the fluctuating velocity ($u^{\prime }_{rms}$) at both 100^∘^ ATDC and 150^∘^ CA as shown in Figs. 7 and 8. Velocity magnitude for all 30 LES cycles are also shown in the background of each figure to illustrate the level of CCV present. The experimental results were an ensemble-average of 100 cycles extracted from [40]. As can be seen from the results below, the numerical predictions using both the RANS and LES approaches discussed above show good agreement with experimental results, generally well predicting variation in mean velocity across the combustion chamber and the magnitude of the fluctuating velocity. Further validation results for the in-cylinder flow field can be found in [41, 42].

The fuel injection model was validated by running the CFD model with the Lagrangian DDM as discussed above, with the LES turbulence model for a further 15 complete engine cycles, and the RANS turbulence model for a further cycle. The ensemble-averaged CFD predictions were compared against experimental results ensemble-averaged across 200 injection events from [43], for: (1) plume tip penetration, (2) plume tip velocity, and (3) D_10_ droplet diameter.

**Fig. 9:**
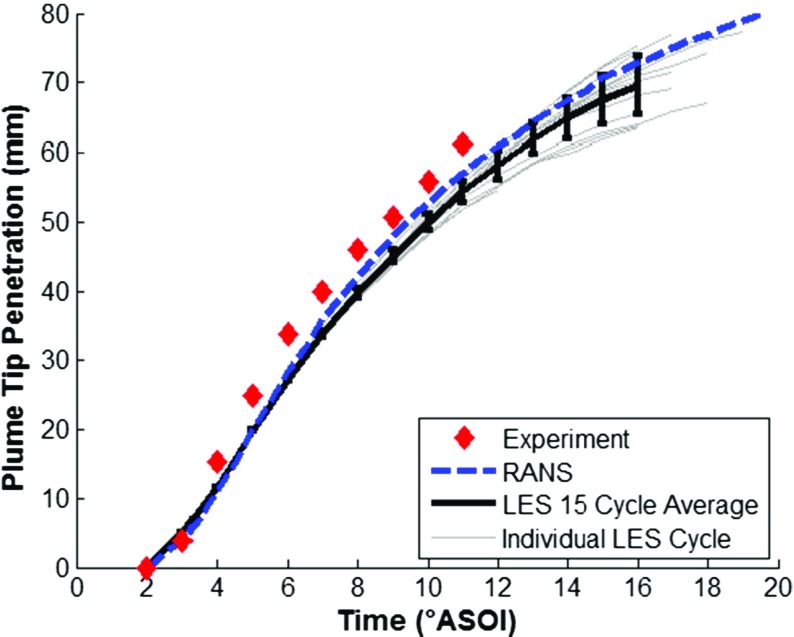
A comparison of experimental [43] and predicted plume tip penetration for iso-octane, showing LES ensemble-average results with error bars for cycle standard deviation, individual LES cycles and RANS predictions

**Fig. 10:**
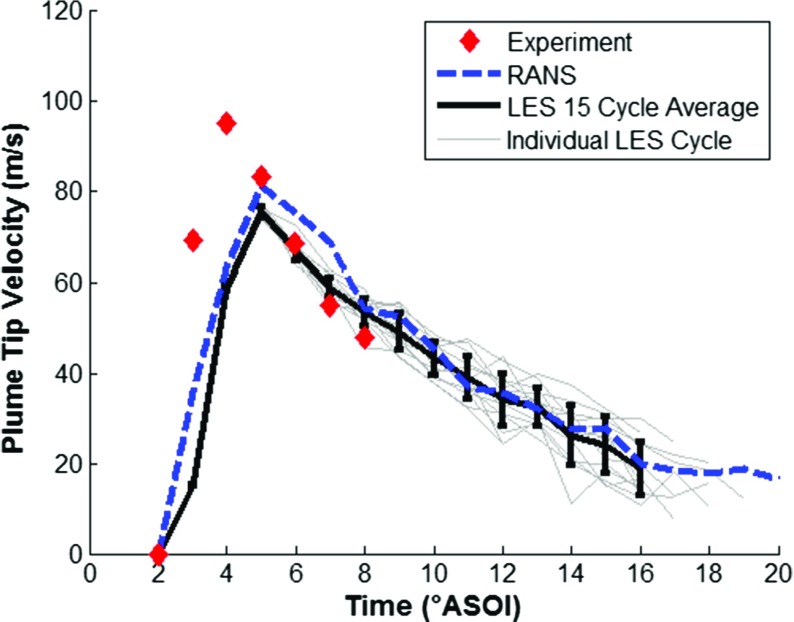
A comparison of experimental [43] and predicted plume tip velocity for iso-octane, showing LES ensemble-average with error bars for cycle standard deviation, individual LES cycles and RANS predictions

**Fig. 11:**
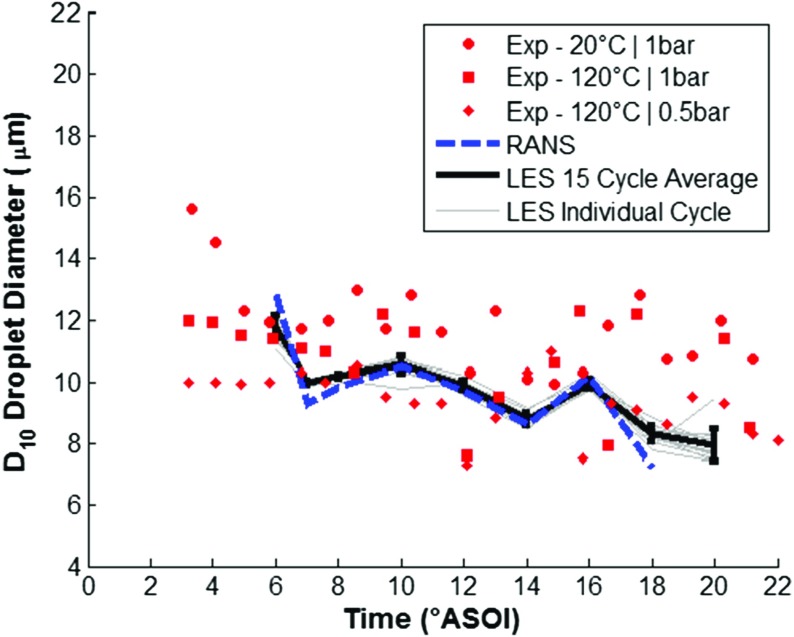
A comparison of experimental [43] and predicted D_10_ droplet diameter at z=-25mm from the injector tip for iso-octane, showing LES ensemble-average with error bars for cycle standard deviation, individual LES cycles and RANS predictions

Figure 9 shows a comparison of experimental and predicted plume tip penetration, with the results indicating relatively good agreement between experimental results and numerical predictions using both LES and RANS turbulence modelling approaches. The spray plume tip velocity was calculated from the derivative of the plume tip penetration and the numerical predictions are compared against experimental results in Fig. 10 showing good agreement. Experimental PDA data of the D_10_ droplet diameter measured in a constant volume chamber at z=-25 mm from the injector nozzle tip across a range of temperature and pressure conditions are compared against the numerical results and shown in Fig. 11. Unfortunately, experimental results were not available at the standardised condition of T_f_ = 363K and 0.5bar gas pressure but the results suggest that the droplet diameter is within the expected range and the change in droplet diameter over time closely matches the experimental results providing increased confidence in the capability of the droplet breakup model to satisfactorily predict the secondary breakup processes. Further validation results for the fuel injection event can be found in [44, 45].

## Results and Discussion

### Characteristics of turbulence intensity

The velocity fluctuations, often referred to as the ‘turbulence intensity’, were first evaluated at spark timing across the cylinder via the use of velocity fluctuation contour diagrams with the cutting plane in the xz-plane or tumble-plane. To do this the velocity fluctuations were calculated as follows:

For the LES predictions, the velocity fluctuations were calculated as follows:

The velocity fluctuations were calculated as defined by Eq. 3.

3 $$ {u}^{\prime}_{i} (\theta ,c)=u_{i} (\theta ,c)-\bar{{u}}_{i} (\theta ) $$

Where *c* is cycle number, *n* is the total number of cycles and *𝜃* the crank angle.

Here, $\bar {{u}}_{i} $ is the ensemble-averaged velocity across all 15 engine cycles and defined by Eq. 4.

4 $$ \bar{{u}}_{i} (\theta )=\frac{1}{n}\sum\limits_{c = 1}^{n} {u_{i}} (\theta ,c) $$

Finally, the RMS velocity fluctuations were calculated as defined by Eq. 5.

5 $$ {u}^{\prime}_{i,rms} (\theta )=\sqrt {\frac{1}{2}\sum\limits_{c = 1}^{n} {{u}^{\prime}_{i} (\theta ,c)^{2}}} $$

Velocity fluctuations from the RANS predictions were calculated by rearrangement of the underlying Boussinesq assumption such that the velocity fluctuations are defined as a function of the turbulence kinetic energy as shown in Eq. 6.

6 $$ {u}^{\prime}_{i} (\theta )=\sqrt {\frac{2}{3}k} $$

As a consequence of the isotropic assumption within the Boussinesq equation, each velocity fluctuations are equal in all three spatial planes.

**Fig. 12:**
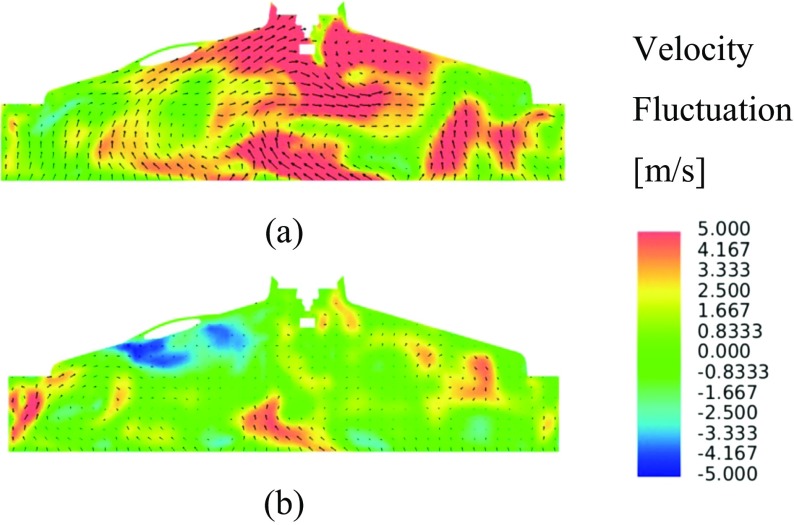
LES velocity fluctuation contours and vectors in the xz-plane intersecting the spark plug electrodes for two arbitrary cycles with **a** high levels of turbulence intensity and **b** low levels of turbulence intensity

Velocity fluctuations for two arbitrary LES cycles are presented along the xz-plane, one exhibiting high levels of velocity fluctuations Fig. 12a, and one exhibiting low levels of velocity fluctuation Fig. 12b. In this cutting plane, significant variation in the both the magnitude of velocity fluctuations and the small scale turbulent structures is evident.

**Fig. 13:**
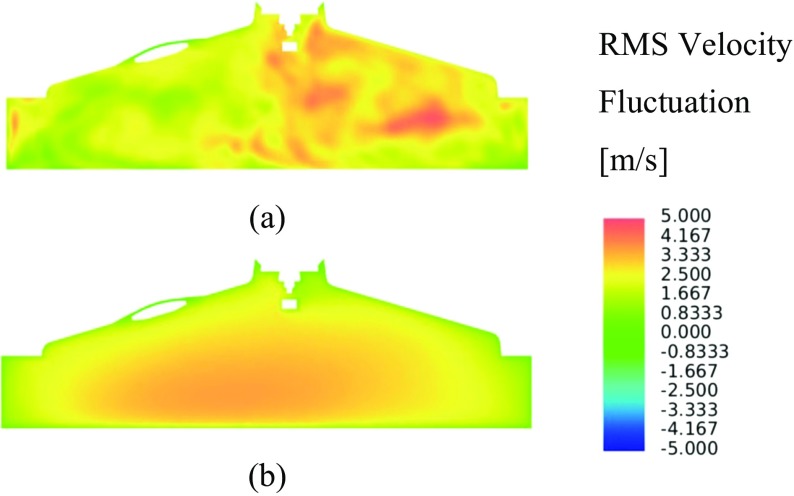
**a** LES RMS velocity fluctuation contours, **b** RANS velocity fluctuation contours, in the xz-plane intersecting the spark plug electrodes

**Fig. 14:**
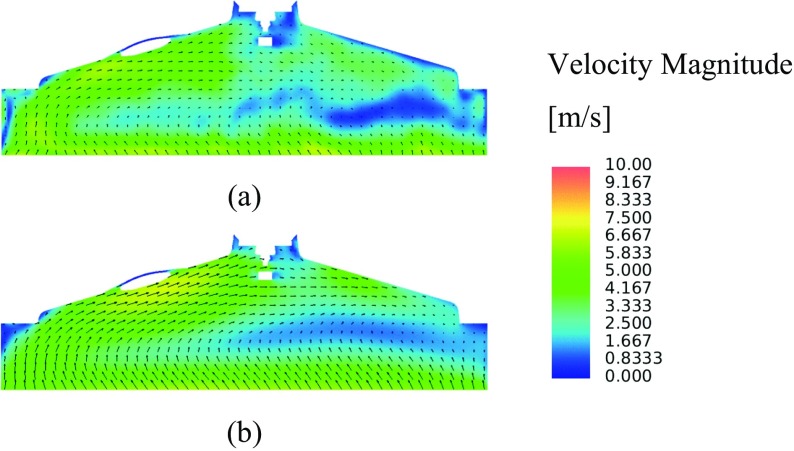
**a** LES ensemble-average velocity magnitude contours and vectors, **b** RANS mean velocity magnitude contours and vectors, in the xz-plane intersecting the spark plug electrodes

Figure 13a shows the LES RMS velocity fluctuation, with higher levels of velocity fluctuations spatially located towards the exhaust side of the combustion chamber as a consequence of the less dominant flow structures characterised by higher levels of small scale turbulence in this area. This becomes obvious by inspection of the ensemble-average velocity magnitude contours shown in Fig. 14a, where a relatively strong clockwise tumble structure is present, but that breaks down on the exhaust side of the combustion chamber as a consequence of the interaction of the flow field with the combustion chamber pent-roof and spark plug electrode geometry.

Figure 13b shows the velocity fluctuations when using the RANS turbulence modelling approach, where, whilst the magnitude of the fluctuations are similar to those in the LES predictions, the effect of the isotropic assumption becomes evident with the spatial variation in fluctuations being poorly represented.

**Fig. 15:**
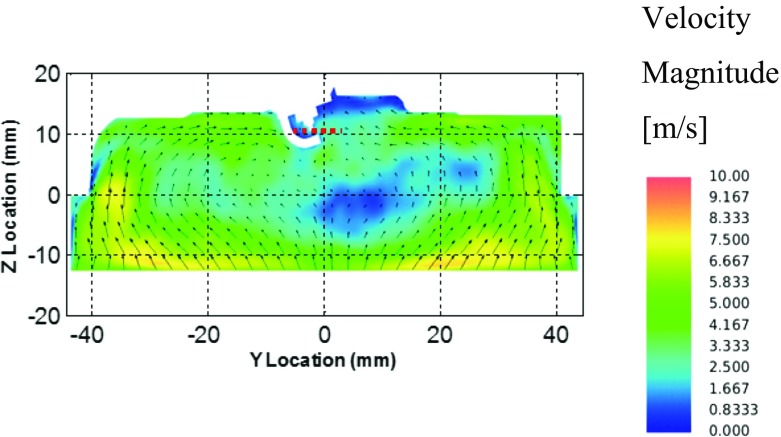
Ensemble-average velocity magnitude contours and vectors in the yz-plane, crossing through the spark plug electrodes

**Fig. 16:**
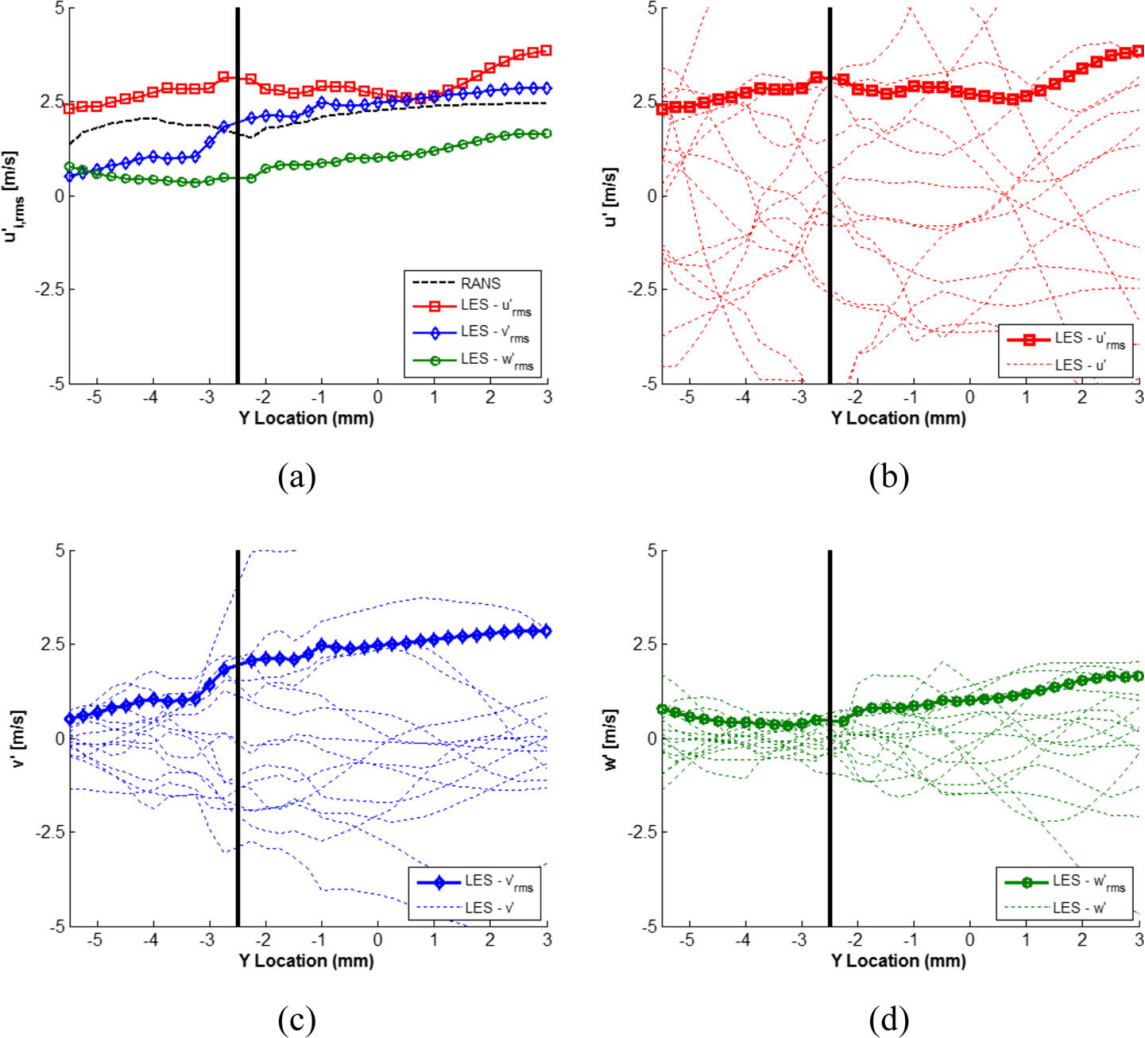
Along the plane [-4,-2.5,10.5], passing through the spark plug electrodes **a** RMS velocity fluctuations for LES and RANS predictions, **b** LES RMS velocity fluctuation and velocity fluctuations for each cycle in the x-plane, **c** LES RMS velocity fluctuation and velocity fluctuations for each cycle in the y-plane, **d** LES RMS velocity fluctuation and velocity fluctuations for each cycle in the z-plane. Note, solid vertical black line in all figures denotes the location of the spark plug electrodes

To investigate the velocity fluctuations in further detail, the fluctuations in each spatial plane were extracted in the yz-plane, in between the spark plug electrodes, to provide further information on how the flame kernel is likely to be influenced by the flow field turbulence at the point of spark ignition. Figure 15 indicates the cutting plane between the spark plug electrodes with a red dashed line, including LES ensemble-average velocity magnitude contours and vectors. Figure 16a shows the LES RMS velocity fluctuations RANS velocity fluctuations and Fig. 16b and d show the LES velocity fluctuations in each spatial plane, between -5.5mm< *y* < 3 where x=-4 and z = 10.5, as indicated by the red dashed line in Fig. 15.

When reviewing the velocity fluctuations in each spatial plane for each engine cycle, Fig. 16b and d, a number of observations can be made.

The relative magnitude of the turbulent fluctuations are greatest in the x-plane and smallest in the z-plane as a consequence of the inherent tumble, swirl and squish flow patterns set up by the intake port and combustion chamber geometry.

Both the fluctuations in the y- and z-planes show a clear increase in magnitude of the velocity fluctuation to the right of the spark plug electrode geometry. As is seen in Fig. 15, in the yz-plane, both clockwise and counter clockwise large scale flow motions are created, with the combined effect of the spark plug electrodes obstructing and reducing the flow velocity of the clockwise eddy to the right of the spark plug electrodes, and the rise in the combustion chamber roof causing a weakening the counter clockwise eddy, causing a subsequent weakening of the large flow motion and an increase in small scale turbulent fluctuations, as seen in the velocity fluctuation plots of Fig. 16c and d.

Also apparent from Fig. 16a, similar to commented previously, whilst the velocity fluctuations of the RANS predictions are of the same magnitude as the LES predictions, they cannot capture the anisotropy present within the velocity fluctuations.

**Fig. 17:**
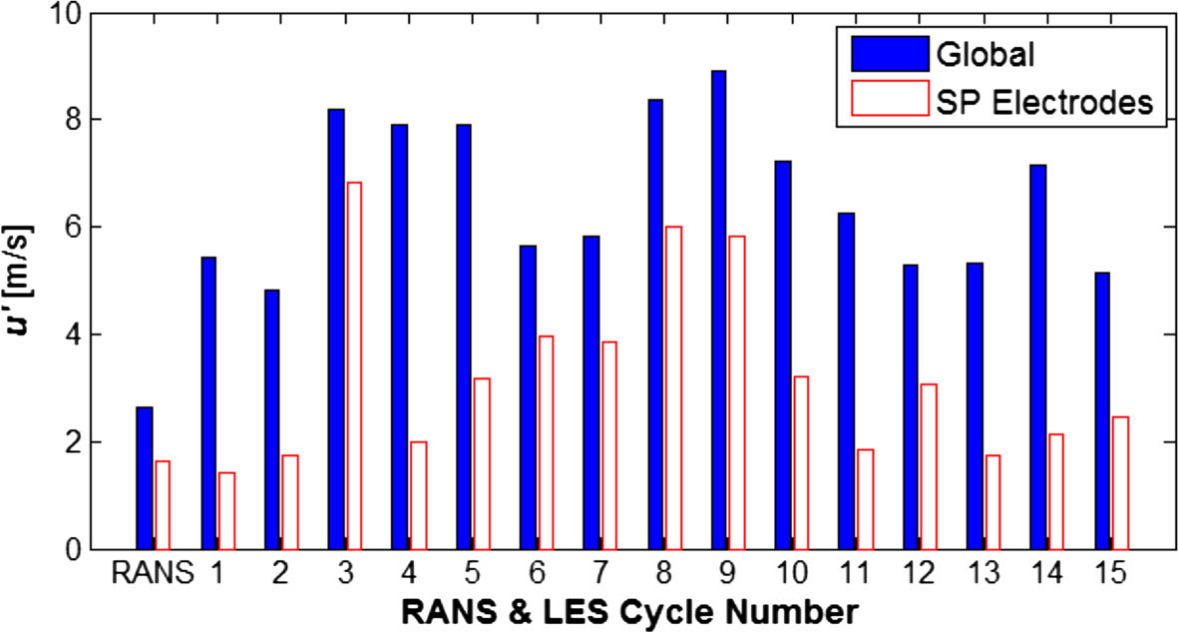
A comparison of the magnitude of velocity fluctuations for RANS and LES predictions (individual cycles), presented as both a global average (solid bars) and at the spark plug (SP) electrodes (hollow bars)

Figure 17 compares the magnitude of the velocity fluctuations when globally averaged and predicted at the spark plug electrodes. Interestingly, the magnitude of the velocity fluctuations near the spark plug electrodes are smaller than when compared to the magnitude of velocity fluctuations seen across the entire combustion chamber. This proves a useful comparison since most experimentally published data typically refers to the RMS velocity fluctuations in the near spark plug region. The works of Malcolm et al. [46], Aleiferis et al. [47] and Rimmer et al. [48], all conducted research on the same engine geometry under investigation here and found RMS velocity fluctuations in the near spark plug region on the order of 3 m/s, 1.5 m/s and 2.25 m/s respectively. This brings confidence to the predicted velocity fluctuations presented here, with the caveat of differences in the size of measurement window between experiments and numerical predictions and the fact that all of the experimental results mentioned have only measured in two spatial planes, rather than the three spatial planes evaluated in these numerical predictions. Values for the velocity fluctuations both near the spark plug and averaged globally, will be compared when investigating the prediction combustion regime later in Section 3.4.

### Regimes of combustion

The regime of combustion expected is predicted using the combustion regime diagrams of [2] and [3]. To achieve this four variables are required to be known; two characteristics of turbulence, the turbulence intensity and the integral length scale of turbulence, and two characteristics of the flame, the laminar flame speed and laminar flame thickness.

The turbulence intensity *u*′ will be taken from the numerical predictions, as presented above, across all cycles using the LES SGS model and for the predictions using the RANS turbulence model. This will show the impact of using turbulence intensity predictions from these RANS and LES turbulence models, and the CCV present across each cycle from the LES turbulence predictions. The combustion regime will also be predicted using turbulence intensity results averaged across the cylinder and results extracted from the region between the spark plug electrodes. This will indicate the relative difference in predicted combustion regime as a consequence of using global turbulence intensity predictions which had a $u^{\prime }_{rms}$ of greater magnitude but with lower CCV, and the turbulence intensity predictions in the near spark plug region which had a lower $u^{\prime }_{rms} $but much greater CCV.

The works of Aleiferis & Behringer [49] conducted on the same engine geometry under investigation here, found the integral length scale of turbulence l_t_ at spark timing in each spatial plane to be: l_u_ 2-5 mm, l_v_ 5-8 mm and l_w_ 3-7 mm, thus giving an approximate integral length scale of 5 mm. This is in agreement with a number of other published works. The early works of Abraham et al. [2] and Fraser & Bracco [50] found that the integral length scale could be approximated as 21% and 10-20% of the distance between the piston and top of the combustion chamber, respectively. Calculating an equivalent height for the pent-roof cylinder head geometry in this study by dividing the cylinder volume by the bore area provides a longitudinal integral length scale using the aforementioned correlations of 4.1 mm and 2-3.9 mm respectively. Heim & Ghandhi [51] also found the longitudinal integral length scale to be in the region of 5-8 mm at TDC in a similar engine configuration, again providing additional confidence in the integral length scale used in this research.

The laminar flame thickness *δ*_*l*_, was approximated as 0.0185 mm for iso-octane, as presented in [49].

The laminar flame speed u_l_, sees significant variation within the published literature due to the difficulty of its measurement and its dependence on a number of variables including charge pressure, temperature and composition, and the fuel-air equivalence ratio. Assuming the predicted in-cylinder conditions of approximately 3 bar, 400K, equivalence ratio of one and a residual gas fraction of 0.18 [52], using the correlation of Metghalchi & Keck [53] and Marshall et al. [54] provides a laminar flame speed for iso-octane of 0.24m/s and 0.20m/s respectively, thus a laminar flame speed of 0.22 m/s was used in this research. Aleiferis, Serras-Pereira and Richardson [52] also approximated a laminar flame speed of 0.20 m/s using combustion imaging results at flame radii less than 1mm in the same engine geometry as presented here, which is in agreement with presented findings from the literature.

**Fig. 18:**
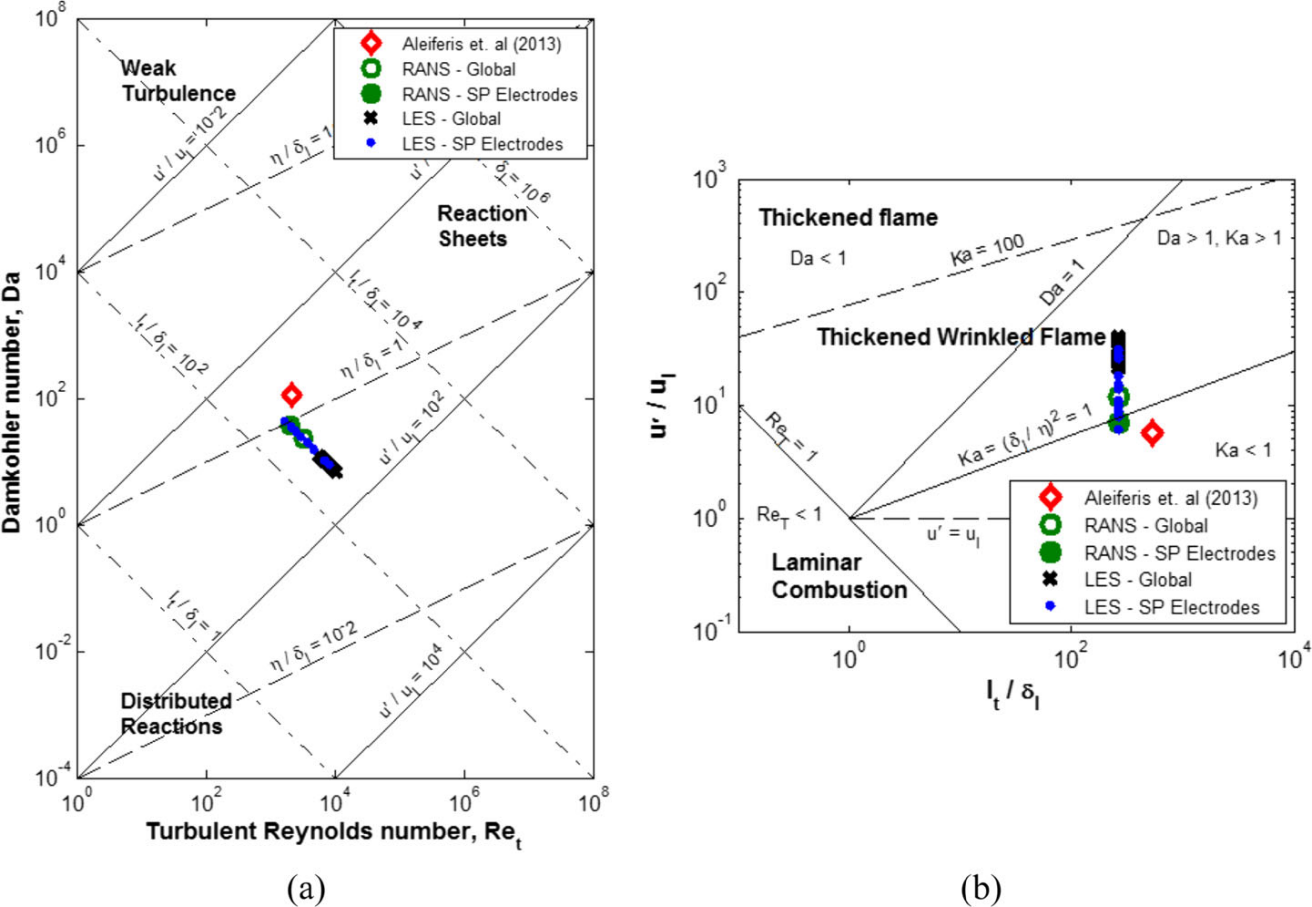
**a** Da-Re_t_ diagram and **b** Borghi diagram, including results from [52] and results for both RANS and individual 15 LES cycles using turbulence intensity predictions both in the near spark plug region and averaged globally

These results are applied to the combustion regime diagrams of [2] and [3], and shown in Fig. 18a & b where a number of observations can be made.

The differences seen between the RANS and LES predictions are as a consequence of the increased velocity fluctuations predicted with the LES turbulence modelling approach. This is to be expected. The premise of LES is that the large scale flow structures are resolved, allowing the large scales to respond to the nonlinear terms present in the momentum equations. This causes an increase in the number of flow structures, kinetic energy and hence velocity fluctuations present within the flow field. RANS turbulence models on the other hand are highly dissipative, reducing the energy present within the flow field and thus the magnitude of the turbulent fluctuations.

An interesting suggestion from these predictions is that for certain cycles (and the RANS predictions) the results fall around Karlovitz number of unity. This indicates that the flame thickness may be smaller than the Kolmogorov scale for certain cycles but for other cycles, and certainly as the flame propagates further into the centre of the combustion chamber, the Karlovitz number will be greater than unity and hence the smallest scales are able to enter the inner flame structure and thicken the wrinkled flame front. With respect to LES combustion modelling, the thickened flame LES (TFLES) combustion model [55] has been proven to well predict combustion across a range of both wrinkled and thickened-wrinkled flame conditions within an ICE and would appear a good choice for modelling the effects of flame thickening during CCV [56, 57]. As expected, in all cases the turbulence intensity is predicted to be greater than the laminar flame speed.

Also of note is that the CCV in the velocity fluctuations near the spark plug electrodes shows almost an order of magnitude variation in *u*^′^/*u*_*l*_ (Fig. 18a) which indicates almost an order of magnitude of variation in the strength of the turbulent flow field relative to the propagating flame front.

The results from [52], conducted on the same engine geometry and operating condition investigation here, are also added to the combustion regime diagrams which shows a similar expected flame structure to the numerical predictions here, with the differences between it and the results presented in this research primarily being due to a smaller turbulence intensity being used (taken from the near spark plug region) and small differences in values chosen for the laminar flame speed and thickness of iso-octane.

## Summary and Conclusions

This paper has presented the use of a 3D-CFD model using both the RANS and LES turbulence modelling approaches, and a Lagrangian DDM to model an early injection fuel injection event, to predict the velocity fluctuations in the cylinder of a GDI engine across 15 engine cycles. The numerical predictions for velocity fluctuations, both near the spark plug electrodes and averaged across the cylinder, were then applied to the combustion regime diagrams of [2] and [3], utilising results for integral length scale and laminar flame speed and thickness from published experimental research. The main conclusions from this work are as follows:

- The velocity fluctuations were found to vary across the cylinder widely from cycle-to-cycle.
- The LES predictions showed higher levels of RMS velocity fluctuations on the exhaust side of the combustion chamber as a consequence of the spark plug electrode and pent-roof geometries generating less dominant flow structures characterised by higher levels of small scale turbulence.
- The RANS predictions showed velocity fluctuations of similar magnitude to the LES predictions but do not capture the spatial variation in fluctuations.
- The velocity fluctuations near the spark plug electrodes were also evaluated due to it providing information on how turbulence is likely to affect early flame development and for comparison against experimental works. The velocity fluctuations were seen to be largest in the x-plane and smallest in the z-plane as a consequence of the large scale flow patterns setup by the intake port and combustion chamber geometries.
- The influence of the spark plug electrodes on increasing velocity fluctuations was clearly visible in y- and z-plane velocity fluctuations.
- The globally averaged velocity fluctuations were found to be of higher magnitude than those seen near the spark plug electrodes but generally exhibited lower levels of cycle-to-cycle variation.
- Comparison to several experimental works on the same engine geometry under study here, showed the predicted velocity fluctuations to be of very similar magnitude.
- Differences between predictions using LES and RANS turbulence modelling approaches were seen in the positioning on the combustion regime diagrams, due to the inherently reduced dissipative effect of the LES approach predicting greater velocity fluctuations.
- The large cycle-by-cycle variation in turbulence intensity near the spark plug electrodes suggested significant variation in the relative strength of the in-cylinder turbulence and resultant thickening of the propagating flame front.
- Results from [52] were also compared to the results presented here and showed a similar expected flame structure.
